# Supplementary material for: Diaphragm thickness and mobility elicited by two different modalities of inspiratory muscle loading in heart failure participants: A randomized crossover study
Source: PLoS One. 2024 May 24;19(5):e0302735. doi: 10.1371/journal.pone.0302735 (PMC11125520; doi:10.1371/journal.pone.0302735)
Supplement: S1 Fig — Shapiro-Wilk test. (DOCX) [file pone.0302735.s001.docx]

**S2 Fig.** Normality test plot of variables. Shapiro-Wilk test.
