# Supplementary material for: Diaphragm thickness and mobility elicited by two different modalities of inspiratory muscle loading in heart failure participants: A randomized crossover study
Source: PLoS One. 2024 May 24;19(5):e0302735. doi: 10.1371/journal.pone.0302735 (PMC11125520; doi:10.1371/journal.pone.0302735)
Supplement: S1 Table — (DOCX) [file pone.0302735.s002.docx]

**S2 Table.** Normality test of variables.

| Shapiro-Wilk test | Tdi Rest | MIP | FVC | S-Index | MEP |
| --- | --- | --- | --- | --- | --- |
| W | 0,9789 | 0,9141 | 0,9174 | 0,9614 | 0,9434 |
| P value | 0,9464 | 0,1176 | 0,1336 | 0,6571 | 0,3608 |
| Passed normality test (alpha=0.05)? | Yes | Yes | Yes | Yes | Yes |
| P value summary | ns | ns | ns | ns | ns |
