# Supplementary material for: Diaphragm thickness and mobility elicited by two different modalities of inspiratory muscle loading in heart failure participants: A randomized crossover study
Source: PLoS One. 2024 May 24;19(5):e0302735. doi: 10.1371/journal.pone.0302735 (PMC11125520; doi:10.1371/journal.pone.0302735)
Supplement: Attachment — Submitted filename: Response to Reviewers.docx [file pone.0302735.s004.docx]

**Comments to the Author**

**1. Is the manuscript technically sound, and do the data support the conclusions?**

The manuscript must describe a technically sound piece of scientific research with data that supports the conclusions. Experiments must have been conducted rigorously, with appropriate controls, replication, and sample sizes. The conclusions must be drawn appropriately based on the data presented.

Reviewer #1: Partly

Reviewer #2: Yes

**2. Has the statistical analysis been performed appropriately and rigorously?**

Reviewer #1: Yes

Reviewer #2: I Don't Know

**3. Have the authors made all data underlying the findings in their manuscript fully available?**

The [PLOS Data policy](https://na01.safelinks.protection.outlook.com/?url=http%3A%2F%2Fwww.plosone.org%2Fstatic%2Fpolicies.action%23sharing&data=05%7C01%7C%7Ce9361cc6681a4237ad2608dbace8f38f%7C84df9e7fe9f640afb435aaaaaaaaaaaa%7C1%7C0%7C638293888750950346%7CUnknown%7CTWFpbGZsb3d8eyJWIjoiMC4wLjAwMDAiLCJQIjoiV2luMzIiLCJBTiI6Ik1haWwiLCJXVCI6Mn0%3D%7C3000%7C%7C%7C&sdata=sRfB%2BXT%2FF%2BPty0HQ9VGPIfy%2Bm7n98eDB1R1GAwwsxKI%3D&reserved=0) requires authors to make all data underlying the findings described in their manuscript fully available without restriction, with rare exception (please refer to the Data Availability Statement in the manuscript PDF file). The data should be provided as part of the manuscript or its supporting information, or deposited to a public repository. For example, in addition to summary statistics, the data points behind means, medians and variance measures should be available. If there are restrictions on publicly sharing data—e.g. participant privacy or use of data from a third party—those must be specified.

Reviewer #1: No

Reviewer #2: Yes

**4. Is the manuscript presented in an intelligible fashion and written in standard English?**

PLOS ONE does not copyedit accepted manuscripts, so the language in submitted articles must be clear, correct, and unambiguous. Any typographical or grammatical errors should be corrected at revision, so please note any specific errors here.

Reviewer #1: Yes

Reviewer #2: Yes

**5. Review Comments to the Author**

Please use the space provided to explain your answers to the questions above. You may also include additional comments for the author, including concerns about dual publication, research ethics, or publication ethics. (Please upload your review as an attachment if it exceeds 20,000 characters).

**Reviewer #1:**

**C1:**  **This study reports the relationships among diaphragmatic thickness, at end-inspiration and end-expiration, diaphragmatic thickening index and mobility via US under two different modalities of inspiratory muscle loading, in two different modalities of inspiratory muscle loading and different load intensities at full-vital capacity maneuvers and the relationship between diaphragmatic thickness with pulmonary function tests in participants with HF. Interesting study but I have several comments to the authors.**

**A1**. We appreciate your effort in reviewing our manuscript, and we also thank you for making the below comments and suggestions.

**C2. Could the authors provide a schema how they measure diaphragm thickness (Tdi, mm), fractional thickness (TFdi, %), and mobility by US that would be helpful to the readers who are not familiar with this topic.**

**A2:** We appreciate your comment. We have incorporated additional information in "Fig. 2. Diaphragmatic Assessment through Ultrasonography," detailing the Ultrasonography assessment, including thickness (Tdi, mm), fractional thickness (TFdi, %), and mobility (Lines 159-166, page 07).

Additionally, we have inserted a reference (Line 171, page 07).

Number 31: Yamaguti WP, Paulin E, Shibao S, Kodaira S, Chammas MC, Carvalho CR. Ultrasound evaluation of diaphragmatic mobility in different postures in healthy subjects. J Bras Pneumol. 2007 Jul-Aug;33(4):407-13. English, Portuguese. doi: 10.1590/s1806-37132007000400009.

**C3. Does the authors have data on the relationships among cardiac parameters (e.g. cardiac-echo parameters), physical activity (e.g. 6MWD and peak VO2) and Tdi, TFdi and mobility?**

**A3.** We thank you for its valuable comment; in fact, we had already analyzed these correlations. We did not find any differences when correlating LVEF (%), VO_2_ peak and VO_2_ max with TFdi, Tdi and Mobility. These correlations are shown below.

Table. Correlation between cardiac US parameters and physical activity and TFdi (%), Tdi and Mobility.

|  | **TFdi (%)** | | **Tdi (mm)** | | **Mobility (mm)** | |
| --- | --- | --- | --- | --- | --- | --- |
|  | **r** | **p** | **r** | **p** | **r** | **p** |
| **LVEF (%)** | -0.17 | 0.495 | 0.18 | 0.484 | -0.23 | 0.361 |
| **VO_2_peak** | 0.24 | 0.349 | -0.04 | 0.851 | -0.41 | 0.097 |
| **VO_2_máx** | 0.04 | 0.859 | 0.34 | 0.173 | -0.23 | 0.356 |

TFdi, Fractional thickness of the diaphragm; Tdi, diaphragm thickness; LVEF, left ventricular eject fraction; VO_2_, oxygen uptake. Spearman’s correlation.

**C4. What is clinical implications? Could the authors provide clinical implications section in discussion?**

**A4.** Thank you for the suggestion for the clinical implications. As suggested, we addition of the information in discussion. Lines 348-355, pages 15 and 16.

**Reviewer #2:**

**C5: The authors reported an ultrasonographic evaluation of the effects of two types of inspiratory muscle training on the diaphragm in HFpEF patients with normal pulmonary function.**
 A5. We appreciate your effort in reviewing our manuscript, and we also thank you for making the below comments and suggestions.

Minor comments

**C6. Specify whether the patients had any prior history that would affect diaphragm function, e.g., after open heart surgery or cardiac ablation etc.**

**A6.** Thank you for your question . The patients enrolled in the study did not undergo open-heart surgery or cardiac ablation procedures, which could potentially affect diaphragmatic function. Fifteen participants were excluded based on the exclusion criteria: smoking (*n*=4), infections disease (*n*=3) and angina (*n*=3) and being uninterested in participating in the study (*n*=5) (Fig.1).

**C7. Page13, Line 286-288: Specify what functional impairment it is. This reviewer cannot determine from the context whether the functional impairment is heart failure or diaphragm.**

**A7.** We appreciate your suggestion. We have revised the phrase to specify the functional impairment (at Line 300, page 14).

**C8. This reviewer feel that the superiority of ultrasonographic assessment of the diaphragm in patients with heart failure compared to respiratory function tests and CPX should be added.**

**A8.** We thank you for comment. We believe that diaphragmatic ultrasound emerges as a complementary technique for assessing the mass, contraction, and function of the diaphragm in individuals with heart failure. We have included additional information about US assessment in the text. (Lines 339- 345, page 15).

6. PLOS authors have the option to publish the peer review history of their article ([what does this mean?](https://na01.safelinks.protection.outlook.com/?url=https%3A%2F%2Fjournals.plos.org%2Fplosone%2Fs%2Feditorial-and-peer-review-process%23loc-peer-review-history&data=05%7C01%7C%7Ce9361cc6681a4237ad2608dbace8f38f%7C84df9e7fe9f640afb435aaaaaaaaaaaa%7C1%7C0%7C638293888750950346%7CUnknown%7CTWFpbGZsb3d8eyJWIjoiMC4wLjAwMDAiLCJQIjoiV2luMzIiLCJBTiI6Ik1haWwiLCJXVCI6Mn0%3D%7C3000%7C%7C%7C&sdata=YnWgsoNJLzwPcj%2BVHNkP59GtjdgrW1SSHqz4SGQJUAs%3D&reserved=0)). If published, this will include your full peer review and any attached files.


If you choose “no”, your identity will remain anonymous but your review may still be made public.


**Do you want your identity to be public for this peer review?** For information about this choice, including consent withdrawal, please see our [Privacy Policy](https://na01.safelinks.protection.outlook.com/?url=https%3A%2F%2Fwww.plos.org%2Fprivacy-policy&data=05%7C01%7C%7Ce9361cc6681a4237ad2608dbace8f38f%7C84df9e7fe9f640afb435aaaaaaaaaaaa%7C1%7C0%7C638293888750950346%7CUnknown%7CTWFpbGZsb3d8eyJWIjoiMC4wLjAwMDAiLCJQIjoiV2luMzIiLCJBTiI6Ik1haWwiLCJXVCI6Mn0%3D%7C3000%7C%7C%7C&sdata=ZeNpRICzMcOrGsriLv1yppe5Kua1nWNWz55jZGamtbY%3D&reserved=0).

Reviewer #1: No

Reviewer #2: No

[NOTE: If reviewer comments were submitted as an attachment file, they will be attached to this email and accessible via the submission site. Please log into your account, locate the manuscript record, and check for the action link "View Attachments". If this link does not appear, there are no attachment files.]

While revising your submission, please upload your figure files to the Preflight Analysis and Conversion Engine (PACE) digital diagnostic tool, [https://pacev2.apexcovantage.com/](https://na01.safelinks.protection.outlook.com/?url=https%3A%2F%2Fpacev2.apexcovantage.com%2F&data=05%7C01%7C%7Ce9361cc6681a4237ad2608dbace8f38f%7C84df9e7fe9f640afb435aaaaaaaaaaaa%7C1%7C0%7C638293888750950346%7CUnknown%7CTWFpbGZsb3d8eyJWIjoiMC4wLjAwMDAiLCJQIjoiV2luMzIiLCJBTiI6Ik1haWwiLCJXVCI6Mn0%3D%7C3000%7C%7C%7C&sdata=7n3RO7m0Dsrnm9f4r8gsvDuPo%2BKSxddInOQXmaM%2BQEU%3D&reserved=0). PACE helps ensure that figures meet PLOS requirements. To use PACE, you must first register as a user. Registration is free. Then, login and navigate to the UPLOAD tab, where you will find detailed instructions on how to use the tool. If you encounter any issues or have any questions when using PACE, please email PLOS at [figures@plos.org](mailto:figures@plos.org). Please note that Supporting Information files do not need this step.
